# Bacteraemia caused by *Lactobacillus casei* in a patient after cardiac surgery. A case report

**DOI:** 10.1186/s13019-023-02334-x

**Published:** 2023-07-12

**Authors:** Aneta Guzek, Paweł Filipowski, Zbigniew Rybicki, Piotr Grabski, Leszek Gryszko, Emilia Sopolińska, Dariusz Tomaszewski

**Affiliations:** 1 Department of Laboratory Diagnostics, Section of Microbiology, Military Institute of Medicine, Warsaw, Poland; 2 Department of Heart Surgery, Military Institute of Medicine, Warsaw, Poland; 3 Department of Anaesthesiology and Intensive Therapy, Military Institute of Medicine, Warsaw, Poland; 4 Centre for Postgraduate Education, Military Institute of Medicine, Warsaw, Poland; 5 Department of Anaesthesiology and Intensive Therapy, Military Institute of Aviation Medicine, Warsaw, Poland

**Keywords:** *Lactobacillus casei*, Bloodstream infection, Implantable cardioverter defibrillator, complications, Cardiac surgery, Case report

## Abstract

**Background:**

Bacteria of the Lactobacillus family are a part of the physiological flora of the oral cavity, gastrointestinal tract, and urinary tract. We have used them in the food industry as probiotics and supplements. In some settings, rod-shaped lactic acid bacteria may become pathogenic. It may occur in immunocompromised or heart disease patients after cardiac surgery, patients with disturbed intestinal wall permeability, or those treated with broad-spectrum antibiotics.

**Case presentation:**

We present rare bacteraemia induced by the probiotic bacterium *Lactobacillus casei* in a 63-year-old patient after the attempted removal of ICD electrodes, complicated by acute regurgitation of the tricuspid valve. The patient underwent urgent cardiac surgery, the electrode elements were removed, and the tricuspid valve was replaced with a biological prosthesis. After surgery, the patient required intensive, multidisciplinary treatment with mechanical ventilation, continuous renal replacement therapy, broad-spectrum empirical antibiotic therapy, parenteral nutrition, and blood product transfusion because of multiple organ failure. On the 14th day of hospitalisation, the clinical symptoms of septic shock were observed. The microbiological investigation was performed, and *Lactobacillus casei* was cultured from a dialysis catheter sample. Dedicated antimicrobials were administered, and the patient was discharged home in good overall condition.

**Conclusions:**

The present case shows that the promoted use of probiotics must be cautiously administered to patients in severe conditions, especially when accompanied by reduced immune system efficiency symptoms.

## Background

*Lactobacillus* spp. are lactic acid bacteria common in the environment, including water, domestic sewage, plants, and human and animal organisms.

In humans, they are a natural component of the bacterial flora in the oral cavity, gastrointestinal tract, and genitourinary system [1, 2]. These bacteria have beneficial effects on the functioning of the human body and are part of everyday functional foods.

Owing to their natural ability to produce lactic acid, they lower the pH of the environment in which they thrive. The industry uses this feature to make fermented milk, yoghurt, cheese, and pharmaceuticals, including probiotics. Lactic acid bacilli are commonly known as probiotic bacteria and belong to “good” bacteria, which play a pro-health role in the body.

We did not consider *Lactobacillus* to be significantly pathogenic. However, it may cause severe infections in patients with immune system dysfunctions. Cases of endocarditis, meningoencephalitis, pneumonia, prostatic joint infection, urinary tract infections, abscesses, or bacteraemia caused by *Lactobacillus* bacteria have been described [3].

## Case presentation

We present the case of a 63-year-old man with second-degree obesity (body mass index, BMI 35.22 kg/m^2^) and chronic coronary syndrome, after a myocardial infarction treated with percutaneous transluminal coronary angioplasty (PTCA) of the circumflex branch of the left coronary artery with stent implantation, and after an episode of cardiac arrest in ventricular fibrillation in 2003. The same year, an implantable cardioverter-defibrillator (ICD)-type VVI was implanted to prevent sudden cardiac death. In 2007, the patient underwent a replacement of ICD with an ICD DDD because the battery was running low. In 2012, a stent was placed in the right coronary artery. Additionally, the patient had hyperlipidaemia and a diaphragmatic hernia.

On November 3, 2021, the patient was admitted to the Interventional Cardiology Unit of the Military Institute of Medicine in Warsaw (WIM) to replace the ICD DDD device because of various episodes of cracking on the electrode, which was interpreted as non-sustained ventricular tachycardia. On November 11 (the second day of hospitalisation), during the ICD removal procedure, a breakdown of the ventricular electrode occurred, with subsequent acute tricuspid valve regurgitation. The patient underwent urgent cardiac surgery; the electrode elements were removed from the right ventricle, and the tricuspid valve was replaced with a biological prosthesis (Edwards Perimount 29 mm, Edwards Lifesciences Corp., USA). After surgery, the patient was admitted to the intensive care unit of the WIM Cardiac Surgery Department. During hospitalisation, the patient required intensive, multidisciplinary treatment with mechanical ventilation, continuous renal replacement therapy, broad-spectrum empirical antibiotic therapy, parenteral nutrition, and blood product transfusion because of multiple organ failure.

Because of the lack of proper heart rhythm, temporary stimulation with pericardial electrodes was used after surgery. On November 25, 2021 (the 23rd day of hospitalisation), an ICD-type DDR was implanted on the right side (clotting of the left subclavian vein occurred).

During hospitalisation in the cardiac intensive care unit, the patient required many modifications of antibiotic treatment: piperacillin-tazobactam and levofloxacin were started as the first antimicrobials. According to our data, pathogens from the species of *Enterobacterales* are the most frequently isolated species. Hence such a choice of therapy. On the fourth day, the patient’s clinical condition deteriorated and biochemical markers of inflammation increased rapidly. The aetiology of the infection was unknown, so two blood samples were collected for culture from the peripheral vein and the central line, and broad-spectrum antibiotic therapy with meropenem and vancomycin was started. The cultures were negative after a five-day incubation period.

**Fig. 1:**
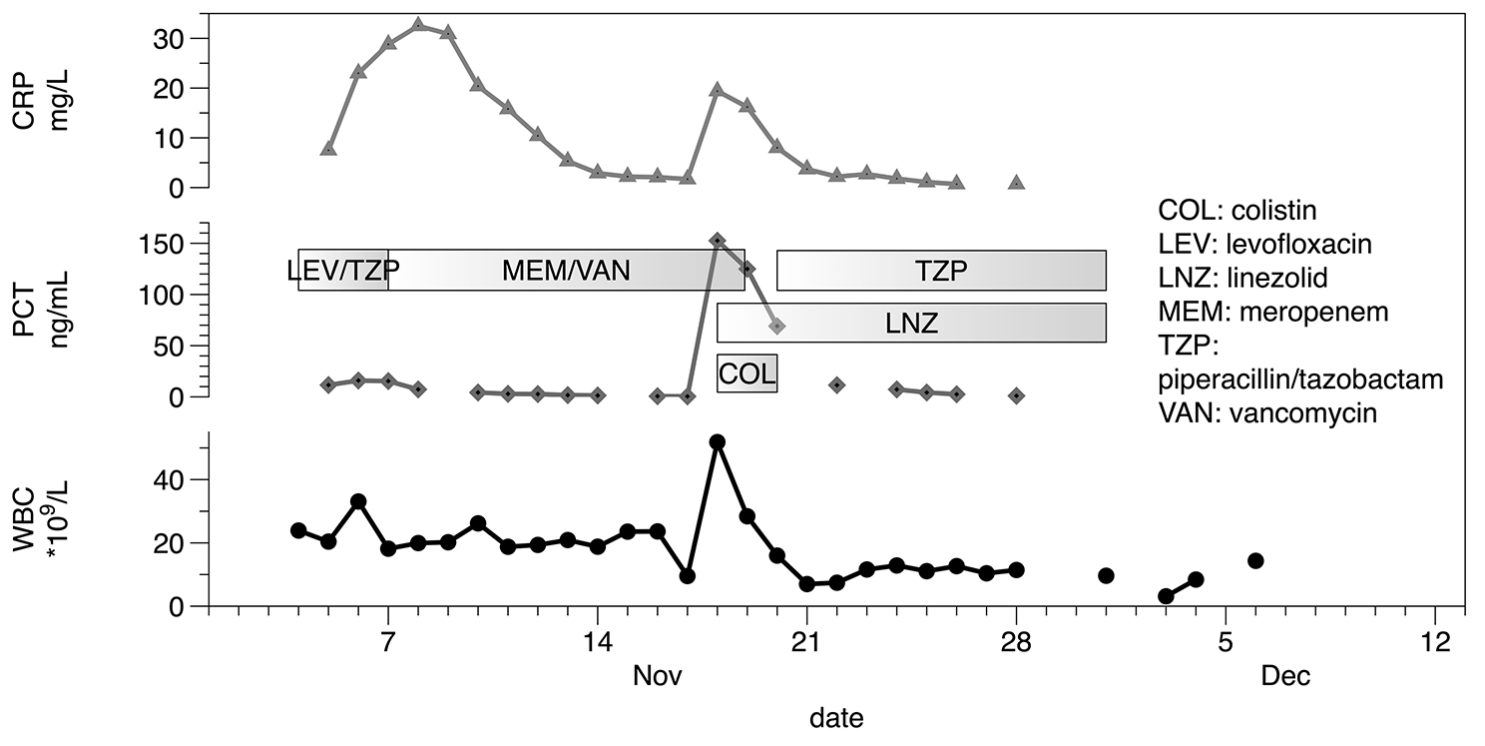
The time course of the serum concentration of the inflammatory markers

On the 14th day of hospitalisation, an increase in body temperature to 39.4 °C was noted, with an increase in inflammatory markers (PCT 155 ng/mL, CRP 19.4 mg/dL, WBC 51.84 × 10^9^/L) and clinical symptoms of septic shock. Additional blood samples were collected for culture from the peripheral vein line, central venous line, and dialysis catheter. Antibiotic therapy was changed again, including empirical administration of linezolid and colistin, because of the suspicion of catheter-associated infection. RT-PCR test was performed for SARS-CoV-2, which was negative. *Lactobacillus casei* was cultured from a dialysis catheter. The antibiotic therapy was changed: colistin administration was ceased, and piperacillin-tazobactam was added to linezolid. The treatment was effective, and the patient’s condition improved with the normalisation of body temperature and inflammatory markers (Fig. 1). Subsequent cultures were negative for pathogenic growth. On November 26, 2021 (24th day of hospitalisation), the patient was referred to the cardiac surgery department for further treatment and rehabilitation because of critical polyneuropathy.

Finally, he achieved a complete return of psychophysical fitness. On December 24, 2021 (52nd day of hospitalisation), the patient was discharged in good overall condition.

It has to be noticed that the lyophilised strains of *L. rhamnosus* (Lakcid Forte® Polpharma SA, Poland) were routinely administered to the patient. Additionally, the patient consumed the Actimel Danone® product, including *L. casei*.

### Microbiologic diagnostics

Blood samples were collected in two BactAlert bottles (BioMérieux, France): a BactAlert FA Plus Bottle to examine aerobes and fungi and a BactAlert FN Plus Bottle for anaerobes. The samples were then placed in BactAlert®Virtuo®, an automatic analyser (BioMérieux, France), and incubated at 37 °C until pathogen growth or the end of the fifth day of examination.

After a 24-hour incubation, the analyser showed a positive result in both bottles. The bottle (BactAlert FA) with positive blood was cultured on a specific growth medium: Columbia agar, McConkey agar, Chocolate agar, and Saboraud agar, while the bottle (BactAlert FN) additionally on Schaedler agar.

The growth media were incubated for 24 h at 37 °C under aerobic or anaerobic conditions. After media incubation, growth of the pathogens was observed on Columbia agar as small α-haemolytic, smooth, opaque, non-pigmented, glistening, and convex with regular edge colonies.

The microscopic investigation found Gram-positive, rod-shaped bacilli that were non-spore-forming.

The isolate was identified as *L. casei* by MALDI-TOF mass spectrometry using Vitek MS (BioMérieux, France). Minimal inhibitory concentration (MIC) values for different antimicrobial agents were determined using Etest® (BioMérieux, France), and the results were interpreted according to the EUCAST-2021 criteria [4].

*L. casei* was susceptible to ampicillin (MIC = 0.5 mg/L), amoxicillin/clavulanic acid (MIC = 0.75 mg/L), linezolid (MIC = 1 mg/L), piperacillin-tazobactam (MIC = 1 mg/L) and resistant to meropenem (MIC = 32 mg/L).

## Discussion and conclusions

Cases of *Lactobacillus* bacteraemia are sporadic. Although lactic acid bacilli cause only 0.1% of the cases [5], the mortality rate is as high as 30% [6]. Analysis of 129 issues of lactobacillemia revealed that the most frequently isolated species were *L. rhamnosus* and *L. casei* [6]. Similar data were presented by the National Public Health Institute in Finland, which showed that more than half (54%) of bacteraemia cases were caused by *Lactobacillus* spp. was caused by *L. rhamnosus*, 19% - *L. fermentum*, and 15% - *L. casei* [7].

Broad-spectrum antibiotic treatment, mainly with vancomycin [6, 8], heart valve defects [9], organ transplant [8, 10], prolonged venous access [5], surgery of the respiratory and digestive tract [10], excessive consumption of milk, dairy products [6, 8], and probiotics [5, 7] may increase the risk of lactic acid bacilli infection. Infections often occur in immunocompromised patients treated with corticosteroids or when the disease reduces the natural immune mechanisms. Moreover, increased vascular permeability and microangiopathy, as seen in diabetes mellitus, may predispose to increased susceptibility to *Lactobacilli* infection [3]. The prophylactic use of probiotics, including *Lactobacillus*, has not been proven effective in preventing lung infections in adults requiring critical care and mechanical ventilation [11].

In the present case, we observed a correlation between these two factors. The first is a severe course of the disease, manifesting as multi-organ failure, and the second is excessive consumption of commercially available food products containing strains of *L. casei*. One can argue that the infection was initiated by the spread *of L. casei* to the lymphatic system in the gastrointestinal tract and later to the bloodstream.

*Lactobacillus* species are usually susceptible to β-lactam antibiotics, carbapenems, and protein synthesis inhibitors. Pathogen isolated from our patient was susceptible to ampicillin, amoxicillin/clavulanic acid, linezolid, piperacillin-tazobactam, and meropenem-resistant. Its resistance to meropenem may correspond to the findings of Anisimova et al. [12] regarding the alarming antibiotic resistance of *Lactobacilli* isolated from commercially available probiotics.

The patient received linezolid for two days before identifying bacteria in the blood, an antibiotic to which the pathogen was sensitive. However, this time was most likely insufficient to prevent growth. Moreover, this antibiotic has bacteriostatic properties, which may not be sufficient to control the rapid growth of the pathogen. Six days of antimicrobial therapy with piperacillin-tazobactam allowed quick elimination of bacteria, reduced inflammation marker levels, and overall clinical improvement.

The presented case shows that probiotics must be cautiously administered to patients in severe conditions, especially when accompanied by reduced immune system efficiency symptoms.

## Data Availability

The datasets used in the current study are available from the corresponding author upon reasonable request.

## Abbreviations

CRP: C-reactive protein
EUCAST: European Committee on Antimicrobial Susceptibility Testing
ICD: Implantable cardioverter-defibrillator
MIC: Minimal inhibitory concentration
PCT: Procalcitonin
PTCA: Percutaneous transluminal coronary angioplasty
RT-PCR: Reverse transcription polymerase chain reaction
SARS-CoV-2: Severe acute respiratory syndrome coronavirus 2
WBC: White blood cells
WIM: Military Institute of Medicine in Warsaw
